# Expression of ribosomal proteins in normal and cancerous human prostate tissue

**DOI:** 10.1371/journal.pone.0186047

**Published:** 2017-10-10

**Authors:** Callum Arthurs, Bibi Nazia Murtaza, Calum Thomson, Kerry Dickens, Rui Henrique, Hitendra R. H. Patel, Mariana Beltran, Michael Millar, Christopher Thrasivoulou, Aamir Ahmed

**Affiliations:** 1 Prostate Cancer Research Centre at the Centre for Stem Cells and Regenerative Medicine, King’s College London, London, United Kingdom; 2 Division of Surgery, University College London, London, United Kingdom; 3 Dundee Imaging Facility, College of Life Sciences, University of Dundee, Dundee, United Kingdom; 4 Department of Pathology, Portuguese Oncology Institute, Porto, Portugal; 5 Department of Pathology and Molecular Immunology, Abel Salazar Institute of Biomedical Sciences, University of Porto, Porto, Portugal; 6 Division of Surgery, Oncology, Urology and Women's Health, University Hospital of Northern Norway, Tromso, Norway; 7 Department of Urology, Princess Alexandra Hospital NHS Trust, Harlow, Essex, United Kingdom; 8 Aquila Biomedical, Edinburgh, United Kingdom; 9 Queen’s Medical Research Institute, University of Edinburgh, Edinburgh, United Kingdom; 10 Research Department of Cell and Developmental Biology, The Centre for Cell and Molecular Dynamics, Rockefeller Building, University College London, London, United Kingdom; University of Alabama at Birmingham, UNITED STATES

## Abstract

Few quantifiable tissue biomarkers for the diagnosis and prognosis of prostate cancer exist. Using an unbiased, quantitative approach, this study evaluates the potential of three proteins of the 40S ribosomal protein complex as putative biomarkers of malignancy in prostate cancer. Prostate tissue arrays, constructed from 82 patient samples (245 tissue cores, stage pT3a or pT3b), were stained for antibodies against three ribosomal proteins, RPS19, RPS21 and RPS24. Semi-automated Ox-DAB signal quantification using ImageJ software revealed a significant change in expression of RPS19, RPS21 and RPS24 in malignant vs non-malignant tissue (p<0.0001). Receiver operating characteristics curves were calculated to evaluate the potential of each protein as a biomarker of malignancy in prostate cancer. Positive likelihood ratios for RPS19, RPS21 and RPS24 were calculated as 2.99, 4.21, and 2.56 respectively, indicating that the overexpression of the protein is correlated with the presence of disease. Triple-labelled, quantitative, immunofluorescence (with RPS19, RPS21 and RPS24) showed significant changes (*p*<0.01) in the global intersection coefficient, a measure of how often two fluorophore signals intersect, for RPS19 and RPS24 only. No change was observed in the co-localization of any other permutations of the three proteins. Our results show that RPS19, RPS21 or RPS24 are upregulated in malignant tissue and may serve as putative biomarkers for prostate cancer.

## Introduction

Prostate cancer (CaP) is the second most diagnosed cancer in men, with an estimated 1.1 million new cases of the disease worldwide in 2012 [1]. In the UK, over 40,000 new cases of CaP were diagnosed in 2014 [2] and an estimated 180,890 new cases were diagnosed in the USA in 2016 [3]. In many countries the death rate from CaP is beginning to decrease [4], however, this is largely being attributed to developments in the treatment rather than the diagnosis of the disease. A biopsy with pathological analysis is the main modality of diagnosis of CaP, with transrectal ultrasound-guided prostate biopsy procedures used as the current method of obtaining a prostate tissue sample [5]. The current diagnostic modalities have drawbacks, such as under sampling, or pathological reporting variation [6]. Gene or protein biomarkers in the biopsy sample could assist in addressing some of these issues. We have previously used largescale tissue arrays to identify proteins that are differentially expressed in non-malignant vs malignant samples using a reproducible and quantitative approach [7, 8], rather than utilising a subjective interpretation of immunohistochemical expression. In the current study, we have applied the quantitative approach to a tissue array containing 245 human prostate tissue samples, gathered from 82 patients, stained for ribosomal (RPS) proteins.

### Ribosomal (RPS) proteins

An increase in ribosome biogenesis is a critical feature of proliferating cells; there is also an increase of ribosomal activity in tumorigenesis [9–11]. The 80S eukaryotic ribosome consists of two subunits: the 60S (large subunit) and the 40S (small subunit) that comprise of both nucleic acid and proteins. The 60S ribosome is thought to be made up of three rRNA chains (25S, 5.8S, and 5S) and 46 protein subunits, whereas the 40S ribosome consists of 18S rRNA and 33 protein subunits [12].

Differential expression or mutations within genes that code for ribosomal proteins are found in malignant tumours of the prostate [13], oesophagus [14], breast [15] and brain [16]. Dysregulation of ribosomal protein mRNA expression has been seen across a range of cancer cell lines [17]. These reports indicate a link between carcinoma and the overexpression of ribosomal proteins such as RPS27. There has been a limited amount of research specifically analysing the relationship between ribosomal protein expression and human CaP tissue. The RPS27 protein is also known to be overexpressed in cancer cell lines [18] which led to RPS27 being proposed as a putative biomarker for CaP [10]. Questions have also been raised about the efficacy of another ribosomal protein, ribosomal protein L19 (RPL19), as a prognostic biomarker following the discovery of differential expression in the gene encoding RPL19 in CaP [19].

Our results show ribosomal protein expression of RPS19, RPS21 and RPS24 is dysregulated in human CaP and increased expression of RPS19 and RPS21 is seen in high Gleason grade CaP that may provide an avenue for developing these as CaP prognostic biomarkers.

## Materials and methods

### Tissue array construction

Details of sample size calculation, patient selection, disease state and tissue block construction have been previously described [7, 20, 21]. Archival, formalin-fixed tissue blocks were constructed using paraffin embedded radical prostatectomy samples gathered from 82 patients between April 1994 and April 2002. All specimens had a preoperative serum PSA level of ≥4 ng/mL and all were pathological stage pT3a or pT3b. All samples were organised into case pairs defined by the following categories: Gleason grade, pathological stage, preoperative PSA concentration. One patient from each pair was considered to have biochemical recurrence (PSA ≥ 0.2 ng/mL-1 within 2 years of surgery), the other was considered to be disease free (undetectable PSA over 3 years after surgery). 6–8μm tissue array (TA) sections were placed onto coated slides which were later diagnosed by a urological pathologist as either benign or malignant (~78% diagnosed as Gleason grade 3+4 or 4+3). Clinical details were then collected and added to a patient spreadsheet.

### Ethics approval

Ethical approval of the use of human tissue for prostate cancer research was granted by the joint University College London/University College London Hospital committees on the ethics of human research. All handling of prostate tissue was carried out in compliance with the International Committee on Harmonization of Good Clinical Practice. TA construction [7, 21] was carried out on archival pathological samples which were later immunohistochemically stained with the approval from, and in accordance with, the University College London/University College London Hospital ethics committees. The ethics review board waived the need for informed consent because archival pathological samples were used.

### Immunostaining of the three RPS proteins in prostate tissue array

RPS19 (ab57643, 500ug/ml), RPS21(ab90874,500ug/ml), and RPS24 (ab102986, 250ug/ml, Abcam, Cambridge, UK) were used in this study either individually (immunohistochemistry, IHC-3,3-diaminobenzidine, Ox-DAB) or in combination (termed multiplex immunofluorescent, IF with FITC, Cy3 and Cy5 fluorophores). For negative controls, primary antibodies were omitted or replaced with IgG matched controls (S1 Fig). RPS19 mouse IgG2b (200ug/ml, 106K4866, Sigma, Dorset, UK) and Rabbit IgG (2ug/ml, ab27472 Abcam, Cambridge) were used with normal IgG protein concentrations as follows: RPS21 0.6ug/ml Rabbit IgG 2ug/ml, RPS24 5ug/ml Rabbit IgG 2ug/ml and RPS19 0.5ug/ml mouse IgG2b 0.5ug/ml. Formalin fixed, paraffin embedded, TA blocks were sectioned at 6–8μm, mounted onto Superfrost plus slides and dried overnight at 60°C. The following protocol was used for tissue staining using the Bond polymer refine detection kit (DS9713, Leica Biosystems, Newcastle, UK). Antigen retrieval was performed using Novocastra pH6 antigen retrieval buffer in a decloaking chamber (Biocare, medical, Pacheco, CA, USA). Sections were heated under pressure for 5 min and allowed to cool for 20 min before equilibrating to ambient temperature under tap water. Alternatively, slides were subjected to the equivalent on-board antigen retrieval using Leica Biosystems, Bond immunostaining robots (Newcastle, UK), using the 20 min ER1 protocol. Following antigen retrieval endogenous peroxidase was blocked using peroxidase blocking solution before incubation with primary antibody for 60 min at RT. Mouse primary (RPS19) sections were then incubated for 15 mins with pre-polymer, followed by HRP polymer for 15 mins before visualisation using Ox-DAB with 10 min washes between incubations using Tris buffered Saline Tween (TBST, 0.05M Tris, 0.85% NaCl, 0.5% Tween pH7.4). Finally, sections were counterstained with Harris’s haematoxylin. Rabbit primary antibodies (RPS21, RPS24) were stained without the use of pre-polymer as this linking reagent is not required when using rabbit primary antibodies.

### Signal quantification

Unbiased signal quantification was carried out using ImageJ software [22] to analyse the number of pixels showing expression compared with the total number of pixels in each converted grayscale image. Quantification of Ox-DAB staining was achieved by first obtaining high resolution images of each tissue core on a bright field microscope (Nikon) for particle analysis with ImageJ, as described previously [20]; imaging tiles of the whole tissue array were also obtained using a Nano-zoomer (Hamamatsu) for illustrative purposes. The amount of tissue present in each image was calculated using a sequence of macros [7, 8] to: open image, invert image, convert image to 16bit, select a predetermined threshold selected using a random training set, convert image to mask, analyse particles (Size 0.5-Infinity, Circularity 0.00–1.00), save then close the image. A similar macro was then constructed to measure the number of particles expressing Ox-DAB signal in the segmented pixels. Amount of signal expressed in each core is given as the amount of Ox-DAB signal / amount of tissue. Particle data (count, total area, average size and area fraction) was then incorporated into a Microsoft Excel spreadsheet containing all TA core data to allow tests of significance of difference between benign and malignant cores and also different Gleason grades (RPS19 Gleason grade 5 (N = 16), grade 6 (N = 11), grade 7 (N = 133), grade 8 (N = 8) and RPS21 grade 5 (N = 13), grade 6 (N = 8), grade 7 (N = 114), grade 8 (N = 9) and RPS24 Gleason grade 5 (N = 19), grade 6 (N = 14), grade 7 (N = 157), grade 8 (N = 10)) using the Mann Whitney U test. Malignant vs benign Mountain plots were constructed using Origin (Microcal) software and area under the curve (AUC) was calculated. Box plots were created in Origin software to illustrate differences in expression between Gleason grades. Medcalc software was then used to create Receiver Operating Characteristics (ROC) curves for statistical evaluation of the protein as a putative CaP biomarker.

### Co-localization of three RPS proteins using immunofluorescence staining

In addition to analysing expression of ribosomal proteins in prostate tissue, co-localization was also calculated as it might be altered in disease. This approach has been described previously [7, 23]. Confocal images of multi-fluorophore, immunofluorescently labelled TA’s were tested for the following antibodies and fluorophores: RPS19 (Cy3 = 559 / 567), RPS21 (FITC, excitation/emission (nm) = 488 / 519), and RPS24 (Cy5 = 635 / 664) using the BondMax system. Individual tissue cores were imaged on an Olympus IX81 confocal microscope with a 40x, 1.3 NA, oil objective lens and 6x digital zoom. Z-section step size was set to 0.17μm, yielding approximately 18–24 z-sections depending on tissue section thickness. Deconvolution of the “oif” images produced was obtained using Huygens Professional software (Scientific Volume Imaging) before quantifying co-localization using the Global Intersection co-localization Coefficient (GIC). GIC can give an intuitive interpretation of the proportion of voxels containing intersecting fluorophore signal vs the number of voxels without intersecting signal. Direct comparisons were made between each single channel image.

## Results

### Ox-DAB signal quantification and analysis

**Fig 1:**
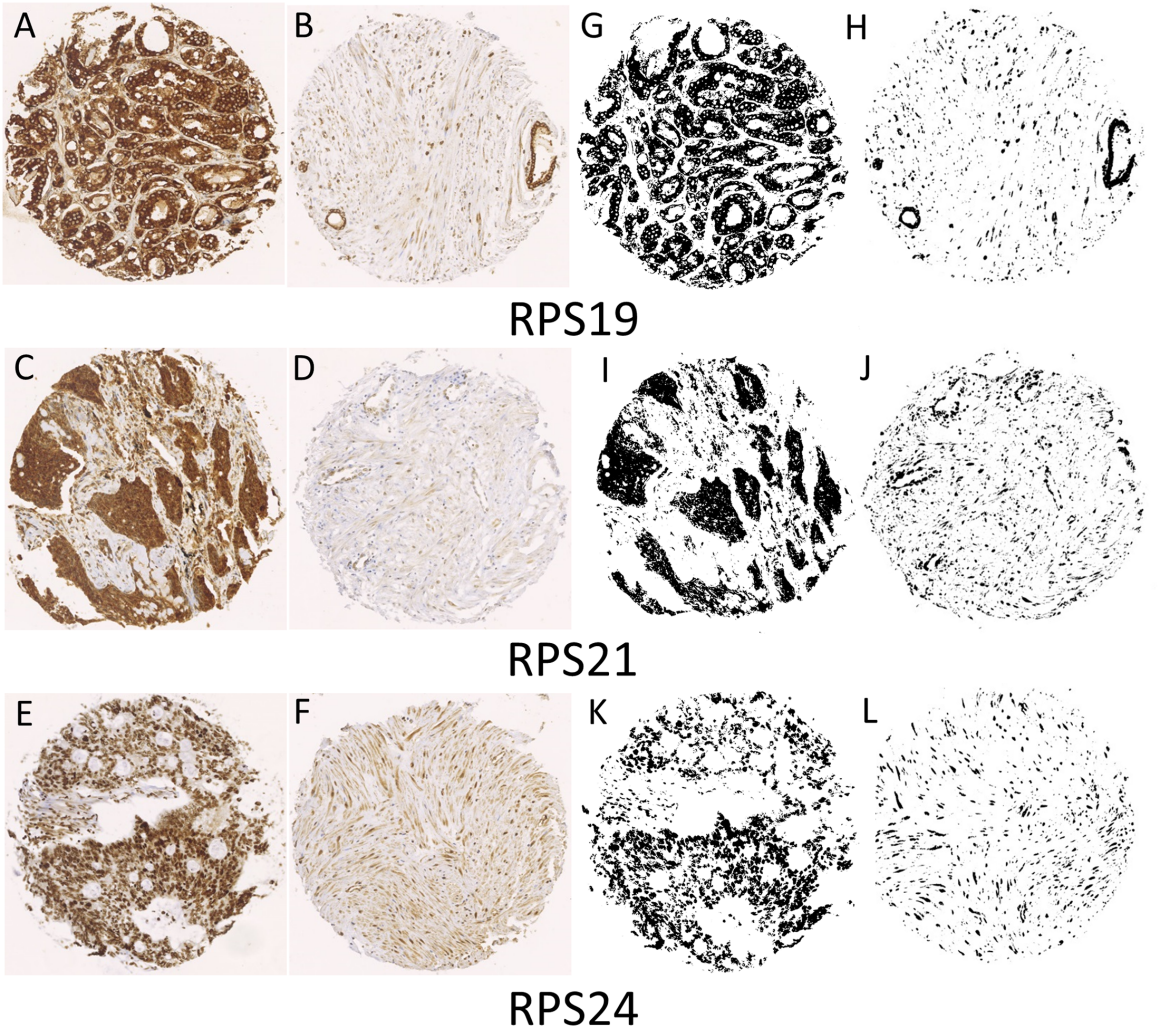
Representative staining for three RPS proteins in malignant human prostate carcinoma tissue (A, C, E) vs non-malignant human tissue (B, D, F). Human prostate tissue samples were obtained from 82 patients and a tissue array was constructed, containing 245 cores, and imaged using a Nikon DXM 1200 digital imaging system with a Nikon Diaphot at 20x magnification and standard bright-field settings. Images of each individual tissue core (3840x3072 pixels) were then used for quantification of the Ox-DAB signal using ImageJ software (see Materials and methods- Signal quantification). Binary, segmented images (G, H, I, J, K, L) represent pixels that were positive for each representative antibody.

**Fig 2:**
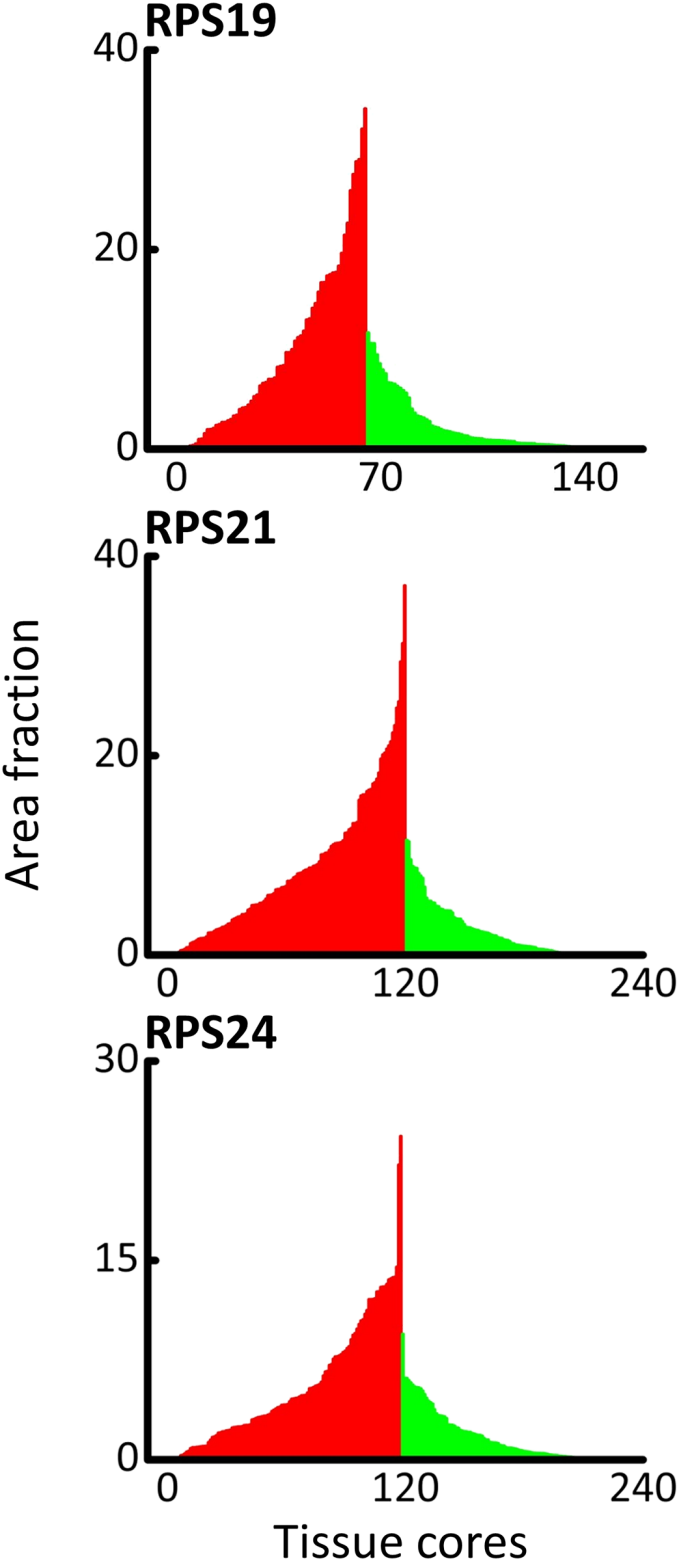
Expression analysis of Ox-DAB signal in prostate tissue. Area fraction (AF) was calculated by dividing the amount of signal, in pixels, by the total number of pixels representing tissue per an unbiased signal quantification protocol (see Materials and methods- Signal quantification). Each bar is representative of the AF of a single TA core. Malignant cores are represented by red and non-malignant cores are represented by green. A significant difference between AUC values was seen for each protein that was tested (p<0.0001).

All RPS proteins showed a generally higher level of expression in malignant cores compared with non-malignant cores (Fig 1). Malignant RPS19 (Fig 1A and 1G) and RPS21 (Fig 1C and 1I) showed largely epithelial expression, as did RPS24 (Fig 1E and 1K) but with the additional observation of RPS24 expression in the nuclei (S2 Fig). Quantitative ImageJ analysis of RPS19, RPS21, and RPS24 showed significant differences between malignant and non-malignant protein expression in CaP (p<0.0001, Mann Whitney U test). These results were incorporated into Mountain plots (Fig 2) so that the amount of signal per amount of tissue in each core for malignant (Red) vs non-malignant (Green) CaP tissue can be compared.

**Fig 3:**
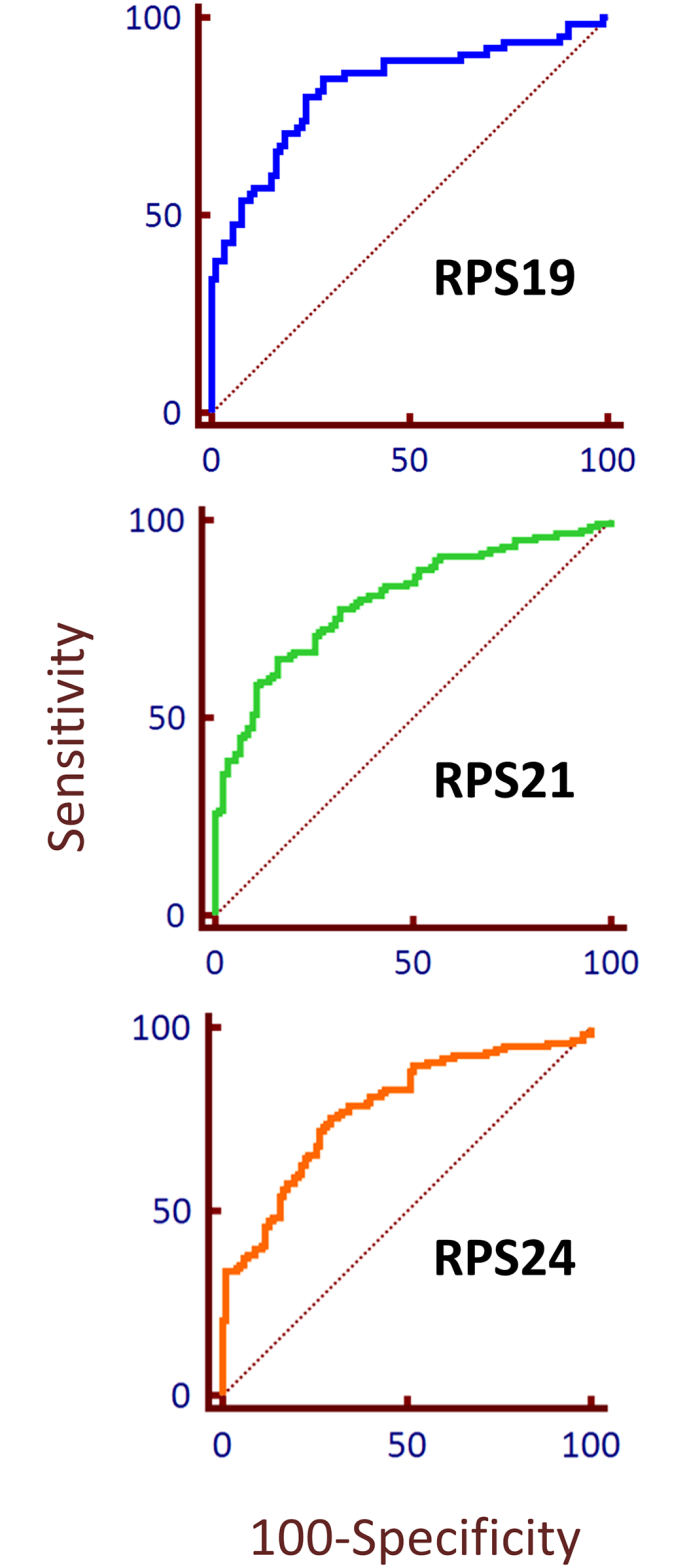
ROC of RPS19, RPS21, and RPS24 in prostate tissue. ROC curve to demonstrate the potential ability of a label to differentiate between malignant and non-malignant prostate tissue. The probit function was used for area fraction per amount of tissue for ROC analysis of RPS19, RPS21, and RPS24. The dotted line is representative of an area under the curve of 0.5 which shows no distinction between the two classifiers (e.g. normal vs malignant).

ROC curves can be used to evaluate the potential efficacy of each protein as a potential biomarker. If the AUC of the ROC curve is <0.5 it is implied that the substance tested would yield no distinction between the two categories. Conversely, the closer the value for AUC is to 1.0, the higher the sensitivity and specificity of the test, and there is a higher probability that the biomarker will correctly identify the disease state of the tissue. Likelihood ratios are used to determine the potential utility of a diagnostic test. A positive likelihood ratio (LR+) was calculated to determine if each ribosomal protein is correlated with the presence of disease. LR+ values over 1.0 suggest that the presence of the measured signal of RPS protein would be linked to the presence of disease. ROC curves were constructed by plotting true positive rate (sensitivity) against false positive rate (100-specificity) for RPS19, RPS21 and RPS24. Area fraction per amount of tissue was converted to probit and fitted to a Gaussian function before incorporation into an ROC curve. LR+ values for RPS19, RPS21 and RPS24 were measured at 2.99, 4.21, and 2.56 respectively, indicating that each of the proteins tested is likely to have been overexpressed because of the presence of disease (Fig 3).

**Fig 4:**
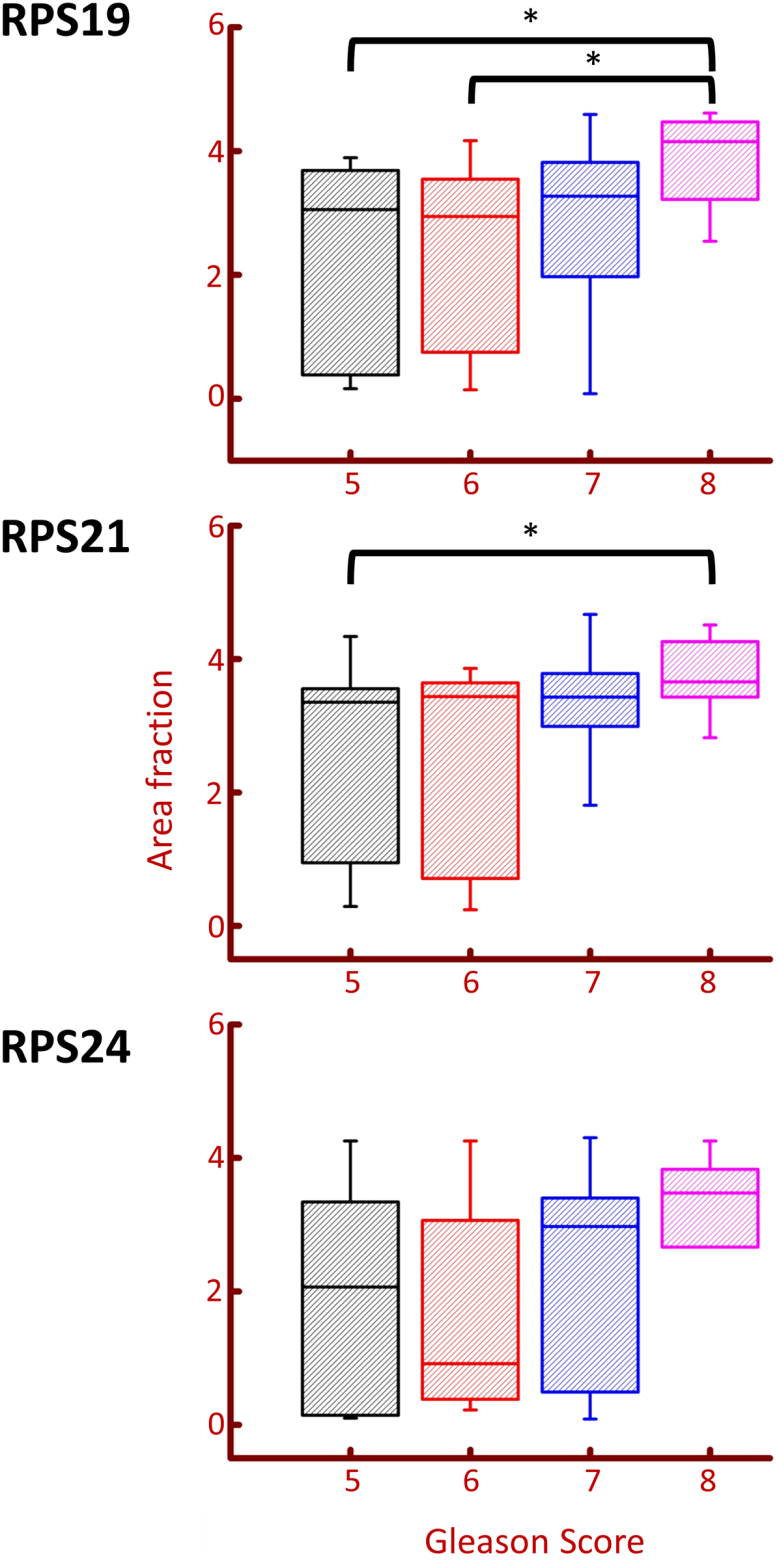
Changes in RPS protein expression with Gleason grade. Box plots were created in Origin software to show the changes in expression of RPS19, RPS21, and RPS24, in different Gleason grades. Brackets represent significant differences in protein expression (p<0.05). A significant change was observed between RPS19 Gleason grades 5 and 8, and 6 and 8 (p = 0.007 and p = 0.028 respectively). A significant difference was also observed in RPS21 Gleason grades 5 and 8 (p = 0.0488).

Changes in RPS19 and RPS21 protein expression were also observed between high and low Gleason grade CaP (Fig 4). RPS19 staining was significantly higher in Gleason grade 8 (N = 8) than it was in both Gleason grade 5 (p = 0.007) (N = 16) and Gleason grade 6 (p = 0.028) (N = 11). RPS21 had significant changes in protein expression for Gleason grade 5 (N = 13) and Gleason grade 8 (N = 9) (P = 0.048). An ROC curve to illustrate the putative use of RPS19 as a marker of high Gleason grade reveals an AUC value of 0.84, which indicates that RPS19 may be a useful protein marker for prognostic purposes.

### Co-localization of RPS19, RPS21 and RPS24

**Fig 5:**
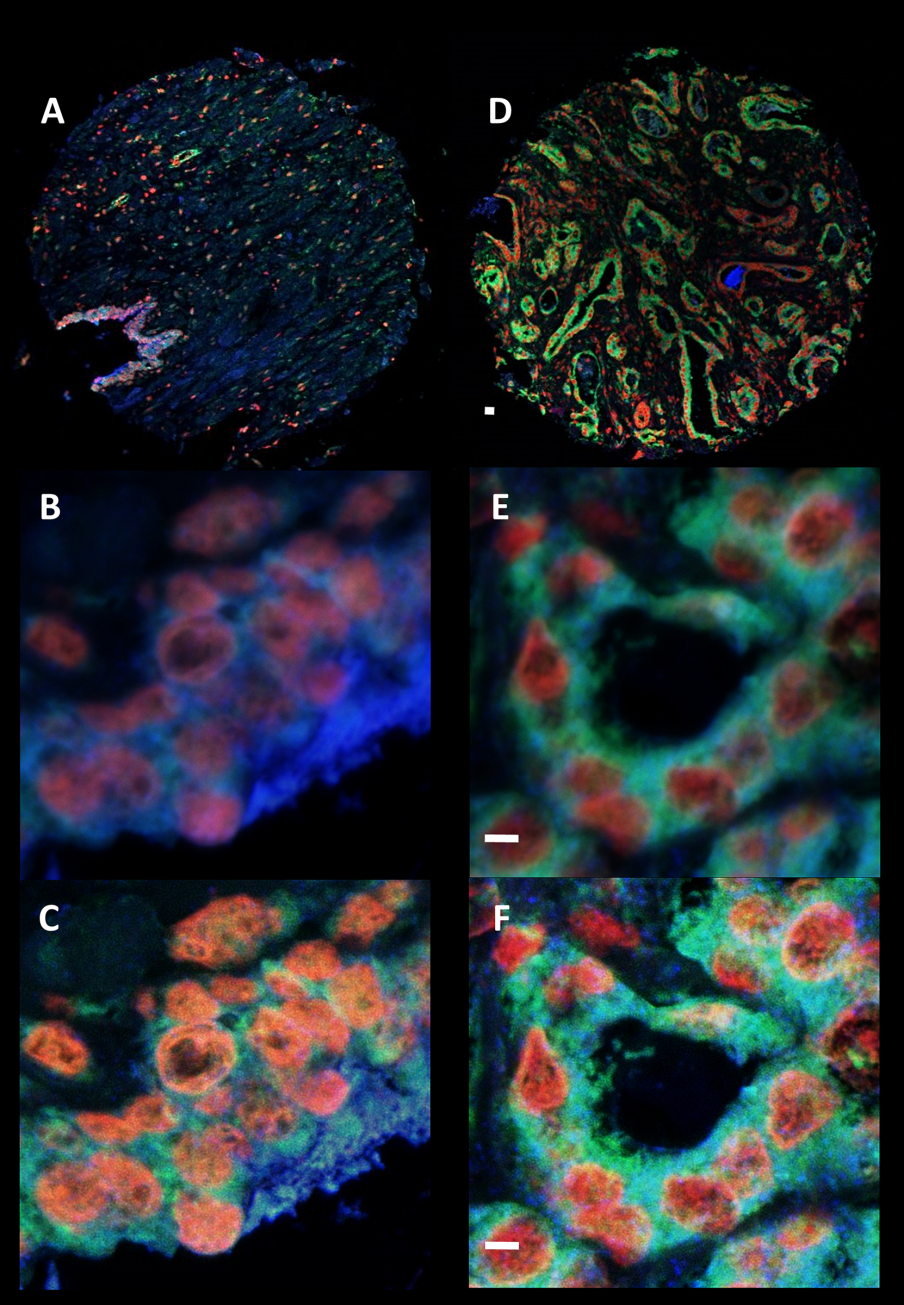
RPS19, RPS21, and RPS24 protein expression using immunofluorescence. Representative images of the co-expression of RPS19 (Cy3- Red), RPS21 (FITC- Green), and RPS24 (Cy5- Blue) which was analysed in both malignant (D, E, F) and non-malignant (A, B, C) prostate tissue. Whole tissue cores (A and D) were imaged on an Olympus IX81 confocal system at low magnification. Intensity of fluorescent signals were optimised at the beginning of each study to prevent oversaturation for each fluorophore. Quantification of co-localization was carried out on images gathered from a randomly selected area of the tissue core using a 40x objective zoom (E is malignant and B is non-malignant) on an Olympus IX81 confocal system. The image was further magnified using a 6x digital zoom. Each image was then deconvolved (C and F) and the GIC was calculated using Huygens software (see Materials and methods). Scale bar = 10 μm.

**Fig 6:**
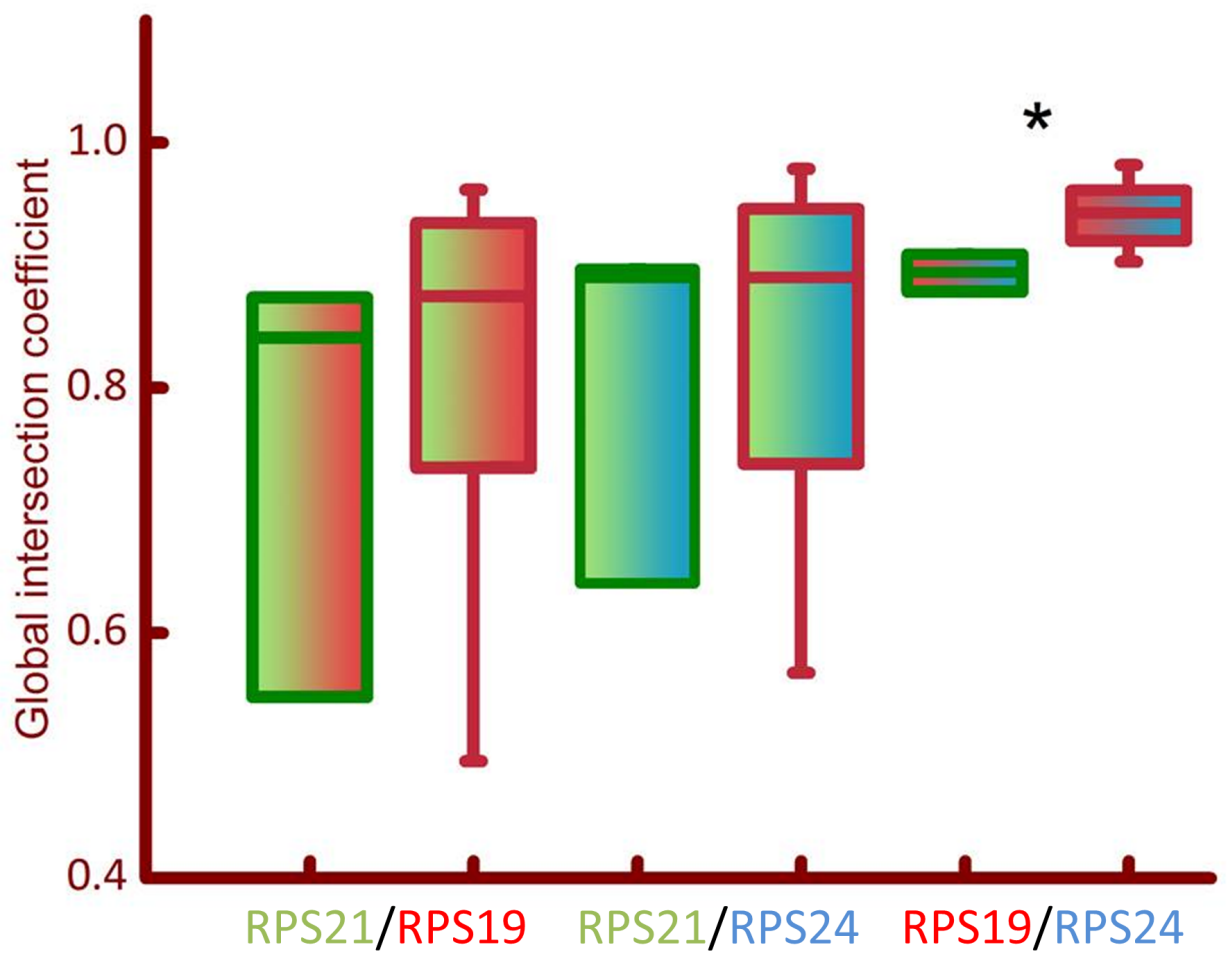
Box plots of co-localization of RPS proteins. Global intersection coefficient (GIC) was calculated using deconvolved, high magnification, fluorescent images (represented in Fig 5) using Huygens software (see Materials and methods). Box plots of GIC were created for comparison between malignant (red bordered bars) and non-malignant (green bordered bars) co-localization for RPS21/RPS19 (green/red bars), RPS21/RPS24 (green/blue bars), and RPS19/RPS24 (red/blue bars). Significance of the difference in GIC values for malignant vs non-malignant prostate tissue was calculated using the Mann Whitney U test (* = *p*<0.01).

Co-localization analysis was used to evaluate changes in the co-expression of two proteins on a TA core (Fig 5). The analysis revealed significant changes in the GIC of RPS19 and RPS24 (Cy3/Cy5) for malignant vs non-malignant CaP tissue (Fig 6). No significant difference was observed between the co-localization of RPS21 and RPS19 (FITC/Cy3) or RPS21 and RPS24 (FITC/Cy5) fluorophore signals. Therefore, changes in co-localization patterns of some ribosomal proteins may facilitate their putative development as biomarkers.

## Discussion

This is the first report describing at least three key proteins of the ribosome complex that are overexpressed in human CaP tissue. Although sparse literature exists regarding the expression of ribosomal genes or proteins in cancer, expression levels of genes coding for ribosomal proteins show large variation across different tissues [24]. Moreover, it appears that the expression of genes involved in ribosomal complex synthesis vary in different cancers [25], which may increase transcription, resulting in the upregulation of a selection of RPS proteins in some cancers. There is no published information about the expression of ribosomal proteins in CaP.

RPS19, a component of the 40S subunit, is mutated in Diamond-Blackfan anaemia [26], a congenital erythroid aplasia characterised by defective erythroid progenitor maturation. A 2005 study found that RPS19 is essential in the development of the 40S ribosomal subunit in *Saccharomyces cerevisiae* [27]. Aside from this, very little is known about the functional role of the protein in the human ribosome. Previous studies have shown that overexpression of *rps19* mRNA is correlated with malignant potential in colorectal cancer (CRC) cell lines. Cell lines such as SW620 and COLO205, which have a malignant pathological character, measured higher expression levels of *rps19* mRNA by northern blot analysis than cell lines associated with a better prognosis (SW480 and COLO201) [28]. There was no analysis into whether the expression of *rps19* mRNA led to a difference in the quantity of RPS19 protein in the cell lines tested. Therefore, the current study may have more relevance by making a post transcription observation into the role of RPS19, with functional consequences relevant to CaP patients. We also show that RPS19 expression is significantly raised in high Gleason grade CaP, which indicates that it may be useful as a putative biomarker of prognosis in CaP.

RPS21 is also a component of the 40S subunit. Little is known about the role of the protein in disease. Expression of RPS21 has previously been measured in prostate samples where approximately 5% of samples exhibited overexpression of the protein (Human Protein Atlas, accessed September 2016). We have shown that RPS21 had significantly raised levels of expression in malignant vs non-malignant CaP tissue although no changes were seen in the co-localization of RPS21 in relation to either RPS19 or RPS24 in CaP. Two somatic mutations (c.62A>G and c.200_217del18) of the *rps21* gene have also been previously identified in a selection of prostate carcinoma samples from the COSMIC database (Cosmic database, accessed September 2016).

The efficacy of RPS24 as a target for diagnosis or treatment of colon carcinoma has previously been evaluated following the discovery that *rps24* knockdown in CRC cell lines (HCT116 and HT-29) reduced cell proliferation and hindered cell migration rate [29]. The study went on to conclude that the *rps24* gene may have a critical role in colon cancer. This raises the question whether RPS24 is also a critical protein in the proliferation and/or migration of CaP cells. This notion is supported by the finding in our study that RPS24 is upregulated in CaP. We have also shown significant changes in the co-localization of RPS24 in relation to RPS19 in CaP which may further support the use of the protein as a putative biomarker. The *rps24* gene has been analysed and published in the Cosmic database, where one somatic mutation in CaP (c.30A>G) from 1306 samples has been identified (COSMIC database, accessed September 2016).

In the current study, we have concluded from quantitative analysis of CaP tissue that a cohort of three ribosomal proteins (RPS19, RPS21 and RPS24) are significantly upregulated in CaP. Furthermore, we have shown that two of these proteins, RPS19 and RPS24 have significant changes in their co-localization patterns in malignant vs non-malignant tissue. Our data suggests that the use of a quantitative approach of measuring protein expression in tissue alongside co-localization analysis with immunofluorescence staining could potentially prove useful in the diagnosis and/or prognosis of CaP.

## Supporting information

S1 Fig Specificity controls. Antibody specificity controls showing representative human prostate tissue stained using Mouse IgG2b at 0.5ug/ml or Rabbit IgG at 2ug/ml and representative images of prostate tissue sections stained following omission of primary antibody used as negative controls to determine background signal. Scale bar 250μm.
(TIF)

S2 Fig Nuclear staining of RPS24. TA slides containing non-malignant (A, B and C) and malignant (E, F and G) CaP tissue cores were stained for RPS24 using an Ox-DAB staining protocol and imaged using a Nano-zoomer (Hamamatsu) slide scanner at 40x magnification. Nuclear staining can be seen in images of both non-malignant (B and C) and malignant (F and G) CaP. Scale bar = 25μm.
(TIF)
