# Supplementary figures and images for: Expression of ribosomal proteins in normal and cancerous human prostate tissue
Source: PLoS One. 2017 Oct 10;12(10):e0186047. doi: 10.1371/journal.pone.0186047 (PMC5634644; doi:10.1371/journal.pone.0186047)

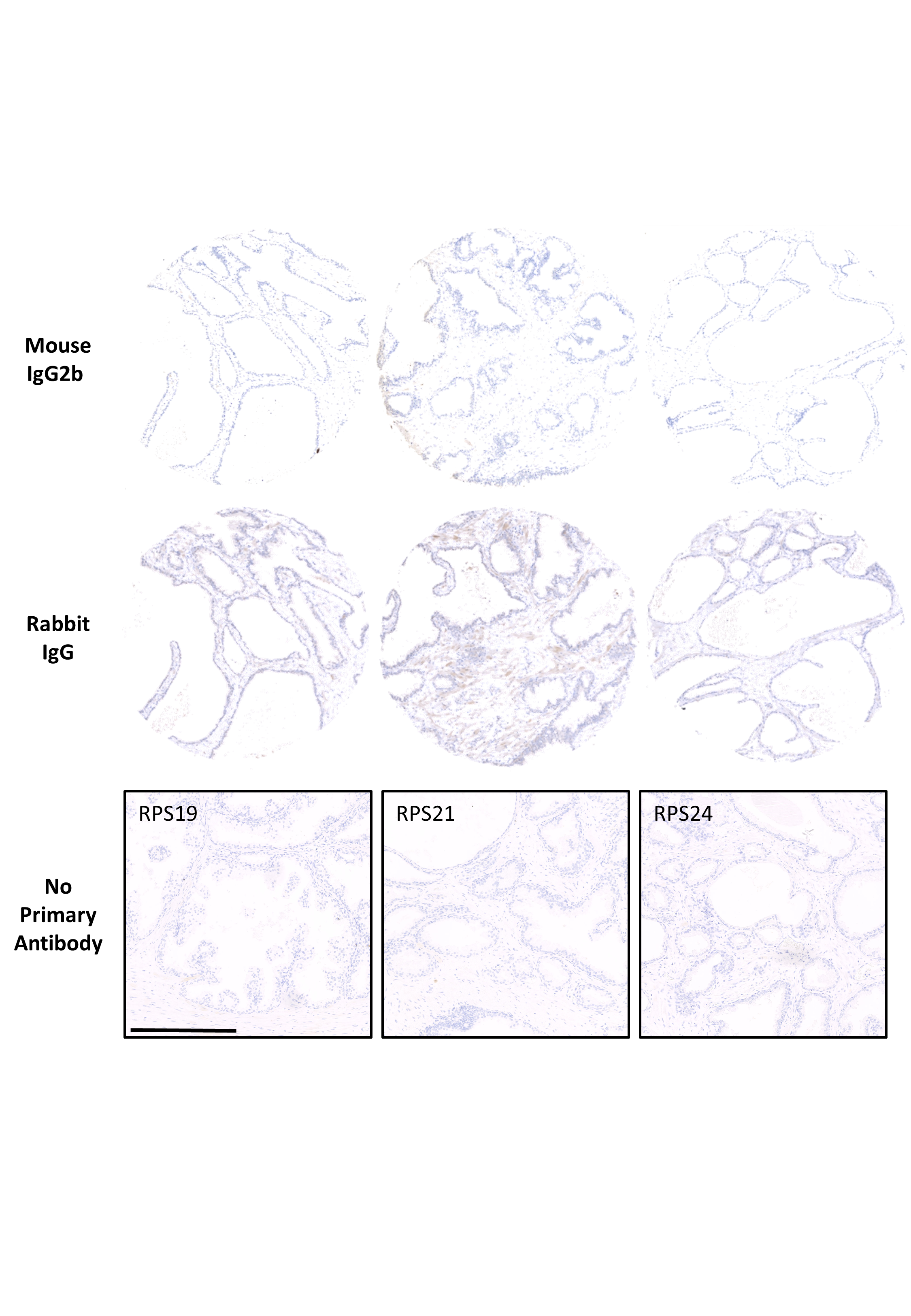

Supplement: S1 Fig — Antibody specificity controls showing representative human prostate tissue stained using Mouse IgG2b at 0.5ug/ml or Rabbit IgG at 2ug/ml and representative images of prostate tissue sections stained following omission of primary antibody used as negative controls to determine background signal. Scale bar 250μm. (TIF) [file pone.0186047.s001.TIF]

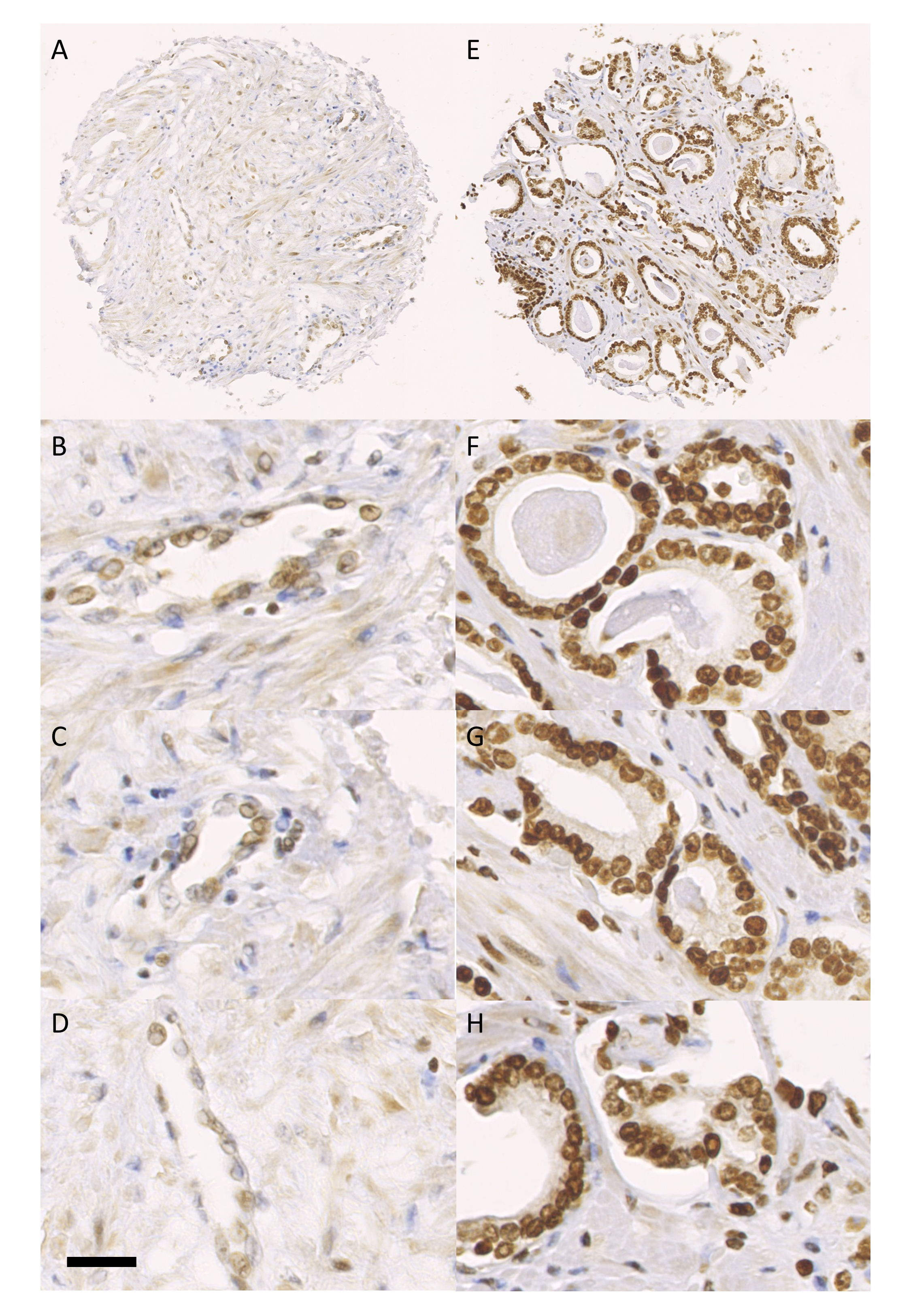

Supplement: S2 Fig — TA slides containing non-malignant (A, B and C) and malignant (E, F and G) CaP tissue cores were stained for RPS24 using an Ox-DAB staining protocol and imaged using a Nano-zoomer (Hamamatsu) slide scanner at 40x magnification. Nuclear staining can be seen in images of both non-malignant (B and C) and malignant (F and G) CaP. Scale bar = 25μm. (TIF) [file pone.0186047.s002.TIF]
